# The radioactive 3D-printed template-assisted CT-guided ^125^I seed implantation for refractory bone metastases: A multicenter retrospective analysis of efficacy, safety, and immune function changes

**DOI:** 10.1371/journal.pone.0347893

**Published:** 2026-05-11

**Authors:** Guang Sheng Zhao, Hou Ze Zhou, Qiu Shi Wang, Xin Yu Zhou, Jin Bo Sun, Fei Gao, Ruo Yu Wang, Zhe Wang, Jun Zhou, Song Liu, Bu Qiang Zhuang

**Affiliations:** 1 Minimally Invasive Interventional Diagnosis and Treatment Center, Affiliated Zhongshan Hospital of Dalian University, Dalian, Liaoning Province, China; 2 Dalian Medical University, Dalian, Liaoning Province, China; 3 Department of Intervention, Dalian Public Health Clinical Center, Dalian, Liaoning Province, China; 4 Department of Intervention, The Second Hospital of Dalian Medical University, Dalian, Liaoning Province, China; 5 Department of Cancer Treatment Center, Affiliated Zhongshan Hospital of Dalian University, Dalian, Liaoning Province, China; 6 Cancer Interventional Center, Linyi Cancer Hospital, Linyi, Shandong Province, China; 7 Department of Interventional Radiology, The Affiliated Hospital of Xuzhou Medical University, Xuzhou, Jiangsu Province, China; Sichuan University, CHINA

## Abstract

**Objective:**

To comprehensively evaluate the efficacy and safety of three-dimensional (3D)-printed template-assisted CT-guided ^125^I seed implantation for refractory bone metastases in a multicenter cohort, to explore its impact on systemic immune function and to identify potential mechanisms.

**Methods:**

This retrospective analysis was conducted on 253 patients with refractory bone metastases who were continuously admitted to Affiliated Zhongshan Hospital of Dalian University, Linyi Cancer Hospital, The Affiliated Hospital of Xuzhou Medical University, Dalian Public Health Clinical Center, and The Second Hospital of Dalian Medical University. The onset time and the duration of pain relief were recorded. The therapeutic efficacy was evaluated by comparing pre- and postoperative Daily Oral Morphine Equivalent Consumption (OMEC), Numerical Rating Scale (NRS) for pain, Quality of Life (QOL) scores, and Karnofsky Performance Status (KPS) scores, as well as by measuring the quantitative changes in target lesion volume and Hounsfield unit (HU) values on imaging before and after the procedure. According to Response Evaluation Criteria in Solid Tumors version 1.1, the objective response rate (ORR) and disease control rate (DCR) were calculated to evaluate tumor response. Flow cytometry was used to detect and compare pre- and postoperative proportions of CD3^+^CD16^+^/CD56^+^ natural killer-like T (NKT) cells and CD3^−^CD16^+^/CD56^+^ NK cells, interleukin (IL)-17A level, and CD4^+^/CD8^+^ T-cell ratio, to assess the changes in host immune function after treatment. Local progression-free survival (LPFS) and overall survival (OS) were calculated, and adverse events were documented.

**Results:**

All patients completed 3D-printed template-assisted CT-guided ^125^I seed implantation, with a procedural success rate of 100%. The clinical symptoms improved after treatment; the median onset time to pain relief was 3.5 days, and the median duration of relief was 10.7 months. The OMEC values and the NRS scores were significantly lower than those before procedure (*P* < 0.05), whereas QOL and KPS scores were significantly higher (*P* < 0.05). The radiologic evaluation showed that ORR and DCR were 42.29% and 69.56%, and 79.05% and 88.93%, respectively, at 1 and 6 months after implantation. The target lesion volumes reduced significantly (*P* < 0.05), whereas the HU value increased significantly (*P* < 0.05), indicating tumor necrosis and osteoblastic repair. The median LPFS was 14.1 months, and the median OS was 19.5 months. The immune function analysis revealed enhanced antitumor immune response, reflected by significant increases in the proportions of NKT cells, NK cells, and CD8^+^ T cells, a decrease in IL-17A level, and a trend toward normalization of the CD4^+^/CD8^+^ ratio (*P* < 0.05). Adverse events mainly consisted of transient local pain of Common Terminology Criteria for Adverse Events grades 1–2. No uncontrollable major bleeding, needle-track seeding, or other severe complication, and no treatment-related death occurred.

**Conclusions:**

The 3D-printed template-assisted CT-guided ^125^I seed implantation was a safe and effective treatment for refractory bone metastases. It provided durable local tumor control and pain relief, and was accompanied by systemic immunological changes. These changes correlated with clinical improvements, suggesting a potential immunological component to the therapeutic effect. This therapy therefore provided a promising treatment strategy for refractory bone metastases.

## Introduction

Bone represents a predominant site of metastasis in patients with advanced malignancies, including lung, breast, prostate, and liver cancers [1–3]. The vertebral column is the most frequently affected anatomical site, followed sequentially by the pelvis, ribs, and femur [4]. Patients with bone metastases typically present with severe localized pain and subsequent functional impairment. In cases of spinal metastases, neurological deficits, such as bowel or bladder dysfunction resulting from spinal cord compression, further compromise both quality of life (QOL) and survival outcomes [5]. Given that bone metastases generally indicate advanced-stage disease, the primary goal of clinical management is palliative care [6,7]. External beam radiotherapy (EBRT) remains the standard-of-care local treatment for painful bone metastases [8]. However, approximately 40% of patients fail to achieve adequate pain relief. This suboptimal response is often attributed to the intrinsic radioresistance of metastatic lesions or anatomical constraints limiting the delivery of therapeutic radiation doses [9].

Interstitial brachytherapy using ^125^I seeds serves as an effective alternative enabling the delivery of precise, high-dose radiation to the tumor lesion while minimizing radiation exposure to surrounding normal tissues [10]. The clinical applications of this technique in bone metastases have demonstrated promising outcomes in terms of local tumor control and pain alleviation [11]. However, the evidence supporting its efficacy in refractory bone metastases, specifically those recurring after EBRT or progressing despite systemic therapy, remains limited. Furthermore, although the immunomodulatory effects of radiotherapy have been increasingly recognized in oncology practice, the potential of ^125^I seed implantation to elicit systemic immune responses in this specific patient population remains poorly understood.

Hence, this multicenter study aimed to comprehensively evaluate two key aspects: first, the local efficacy and safety of three-dimensional (3D)-printed template-assisted CT-guided ^125^I seed implantation (a technique designed to enhance procedural accuracy); second, the impact of this intervention on systemic immune regulation in patients with refractory bone metastases. We hypothesized that this therapeutic approach would provide durable symptom control, and also potentially induce beneficial immunomodulatory effects, thereby offering a novel therapeutic perspective for this clinically challenging patient cohort.

## Methods

### Study population

This was a multicenter retrospective cohort study on patients with refractory bone metastases who underwent 3D-printed template-assisted CT-guided ^125^I seed implantation between June 2019 and June 2024. The definition of “refractory” was established in accordance with relevant clinical expert consensus guidelines to ensure the homogeneity of the study cohort. The study was approved by the Ethics Committee of the Affiliated Zhongshan Hospital of Dalian University, Linyi Cancer Hospital, The Affiliated Hospital of Xuzhou Medical University, Dalian Public Health Clinical Center, and The Second Hospital of Dalian Medical University. All methods were performed in accordance with the relevant guidelines and regulations. All the patients participate in the study have signed the informed consent

### Inclusion criteria

The inclusion criteria were as follows: (1) pathologically confirmed primary malignancy with radiologically confirmed bone metastases, (2) presence of bone metastasis-related symptoms (e.g., VAS score ≥4) attributable to the target lesion, with refractory status defined per clinical consensus criteria (insufficient response to standard drug therapy and unsuitability for or failure of prior radiotherapy), (3) an expected survival of ≥3 months (KPS ≥ 60 or ECOG PS ≤ 2) to allow for follow-up.

Additionally, patients were required to meet the core refractory mandatory criteria as follows: (1) insufficient response to standard drug therapy (analgesics and/or bone-modifying agents for ≥2 weeks, with <30% reduction in VAS score or disease progression), and (2) unsuitability for or failure of prior radiotherapy. At least one of the following was also required: unsuitability for or failure of surgery, ablation, PVP/PKP, or prior non-3D-guided seed implantation.

### Exclusion criteria

The exclusion criteria were as follows: (1) active infection, (2) uncorrectable coagulation dysfunction, (3) pathologic fracture with complete spinal cord injury, (4) severe psychiatric disorders, (5) concurrent local/systemic antitumor therapy, and/or (6) incomplete clinical data.

The study protocol was approved by the institutional review board of each participating center. Written informed consent was obtained from all patients prior to the ^125^I seed implantation procedure.

### ^125^I seed implantation technology

The materials, equipment, and specific methods were the same as those previously reported in our past study [12]. According to the clinical application guidelines for tumor radioactive seed implantation therapy, imaging examination was completed using computed tomography (CT), x-ray, magnetic resonance imaging (MRI), and positron emission tomography (PET)-CT within a week before treatment. Based on the CT scan results, the prescribed dose target was delineated at 0.5–1.0 cm larger than gross tumor volume. A particle implantation treatment planning system, jointly developed by Beijing University of Aeronautics and Astronautics and Beijing Astro Technology Co., Ltd., was used to calculate the total required radioactivity and the number of seeds for each lesion. The corresponding seeds were then procured, and a preoperative plan was formulated through sequential delineation of the clinical target volume and planning target volume (PTV). At the same time, the adjacent risk organs of the tumor were delineated and protected, in which the spinal tumor was > 1 cm from the spinal cord and the dose constraint for the spinal cord was set to a maximum point dose (*D*_max_) of less than 45 Gy, thereby ensuring safety. The prescription dose was 100–120 Gy according to the history of radiotherapy and the conditions of the surrounding organs. The dose was prescribed to the periphery of the PTV, typically corresponding to the 100% isodose line. Dose-volume histograms (DVHs) were generated for each plan, with the goal to deliver the prescription dose to at least 95% of the PTV.

Local and intravenous anesthesia were administered, and laser localization lines were marked according to the body surface. After installing the positioning navigation instrument and 3D template, the insertion position and the angle of the needle were controlled, and the needle insertion channel was established according to a preoperative plan. Starting from the central plane of the tumor, the needles were arranged in layers, with a lateral margin of 1 cm and a depth extending 0.5 cm beyond the distal edge. The seeds were positioned more than 1 cm away from the skin to avoid damage to the skin. If necessary, intraoperative plan modifications and target dose optimization were also performed. Additional parameters were input into the BTPS for seed reconstruction and postoperative dose verification.

### Postoperative treatment and observation

Postoperative follow-up assessments were systematically conducted for all patients, encompassing clinical efficacy, radiographic response, immune function, survival outcomes, and safety. The clinical efficacy was evaluated at 1, 4, 8, 12, and 24 weeks after the procedure using the Numerical Rating Scale (NRS) for pain, Karnofsky Performance Status (KPS) score, QOL score, and Daily Oral Morphine Equivalent Consumption (OMEC). The time to pain relief and its duration were also recorded. Radiographic assessments via CT or MRI were performed at 1, 3, and 6 months after surgery. Tumor response was evaluated according to Response Evaluation Criteria in Solid Tumors (RECIST) version 1.1 to determine the objective response rate (ORR) and disease control rate (DCR), with quantitative analysis of the changes in target lesion volume and Hounsfield unit (HU) values. Tumor density was quantitatively assessed by measuring the mean HU value on noncontrast CT scans. All scans were performed using a standardized protocol on a CT scanner. For each target lesion, a board-certified radiologist blinded to the clinical outcomes manually delineated a two-dimensional region of interest (ROI) along the inner margin of the lesion on three consecutive axial slices best demonstrating the largest tumor diameter. Care was taken to exclude adjacent normal cortical bone, obvious sclerotic rims, and artifacts. The mean HU value for each ROI was automatically calculated using the PACS workstation. The final mean HU value for a given time point was calculated as the average of the three slice-based measurements. Immunological parameters, including the proportions of CD3 ⁺ CD16 ⁺ /CD56 ⁺ natural killer T (NKT) cells and CD3 ⁻ CD16 ⁺ /CD56 ⁺ natural killer (NK) cells, CD4 ⁺ /CD8 ⁺ T-cell ratio, and serum IL-17A levels, were analyzed using flow cytometry on peripheral blood samples collected at baseline (preoperative) and 1 month and 6 months after surgery [13]. The survival analysis included overall survival (OS), local progression-free survival (LPFS), survival rates at various time points, and bone metastasis-specific mortality. Adverse events were documented and graded according to the Common Terminology Criteria for Adverse Events (CTCAE) version 5.0, with particular attention to local pain, neurological symptoms, and serious complications. All follow-up data were censored as of August 2025 or at the date of patient death.

### Statistical analysis

Statistical analysis was performed using SPSS 20.0 and R 4.5.1 software. Continuous data were presented as the mean ± standard deviation or median (interquartile range), as appropriate. Categorical data were presented as frequencies (percentages). For longitudinal comparisons of repeated measures over time (e.g., pain scores and immune parameters), linear mixed-effects models or repeated-measures analysis of variance (ANOVA) with Greenhouse-Geisser correction were used, followed by *post hoc* pairwise comparisons with Bonferroni adjustment. The Student’s *t-*test or Mann-Whitney *U* test was used for between-group comparisons of cross-sectional data. The chi-square or Fisher’s exact test was used for categorical data. Pearson’s or Spearman’s coefficient was used for correlation analysis. Effect sizes (e.g., Cohen’s *d* and *η*²) and 95% confidence intervals (CIs) were reported alongside *P* values where applicable. Survival analysis was performed using the Kaplan-Meier method, with the log-rank test for comparisons. *P* < 0.05 indicated a statistically significant difference.

## Results

### General information

Between June 2019 and June 2024, 253 consecutive patients with refractory bone metastases who underwent ^125^I seed implantation were recruited from the Affiliated Zhongshan Hospital of Dalian University, Linyi Cancer Hospital, The Affiliated Hospital of Xuzhou Medical University, Dalian Public Health Clinical Center, and The Second Hospital of Dalian Medical University (176 men and 77 women). The age of the patients ranged from 36 to 81 years (mean age: 61.8 ± 11.3 years). All patients voluntarily agreed to this treatment modality and signed the informed consent form for seed implantation. This study was approved by the Institutional Review Boards (IRB) of the respective centers. Detailed baseline demographic and disease characteristics of the study cohort are presented in Table 1.

**Table 1:** Clinical baseline information of 253 patients with bone metastases.

Clinical Features	Numerical value
Age	61.83 ± 11.34
Gender	
Male	176
Female	77
Primary tumor site	
Lung	61
Liver	50
Large intestine	33
Prostate	28
Mammary gland	44
Bladder	17
Oral cavity	11
Other	11
Bone metastasis site	
Skull	28
Vertebrae	99
Pelvis, sacrum	61
Ribs	44
Extremity bones	22
Previous treatment	
Radiation therapy	91
Bisphosphonate treatment	135
Untreated	27
Maximum tumor diameter (cm)	
<3	10
≥3	243
Number of tumors	
1	187
2	39
3	27
Tumor characteristics	
Osteolytic	187
Osteogenic	22
Mixed	44

### Treatment feasibility and clinical outcomes

**Fig 1:**
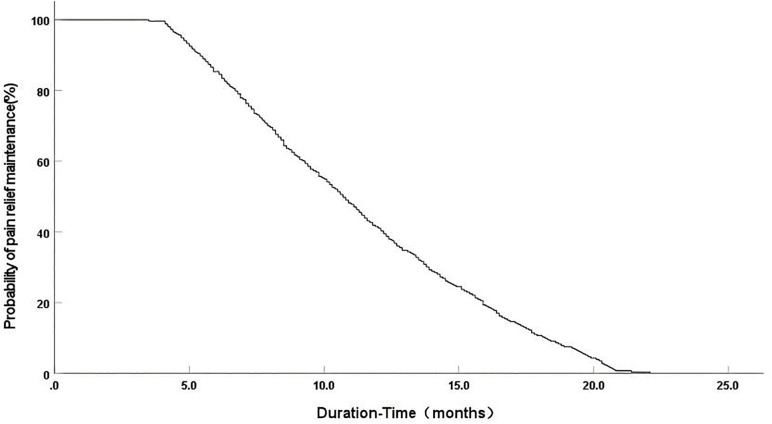
Kaplan-Meier curve of pain relief duration‌‌.

All 253 patients successfully underwent 3D-printed template-assisted CT-guided ^125^I seed implantation, yielding a technical success rate of 100%. The procedure was well tolerated with a mean operative time of (33.3 ± 6.8) min. All procedures were performed under local anesthesia (lidocaine 1%) combined with conscious sedation (intravenous fentanyl and midazolam) as needed. The median postoperative hospital stay was 2 days (range: 1–5 days). Readmission within 30 days due to procedure-related complications occurred in three patients (1.2%), all for managing increased but controllable local pain. Pain relief was rapidly achieved with a median time to onset of 3.5 days (95% CI: 3.2–3.8) and a median duration of 10.7 months (95% CI: 9.8–11.6) (Fig 1).

### Clinical efficacy and survival outcomes

Patients demonstrated significant clinical improvement during the 24-week follow-up. Daily OMEC decreased from (116.67 ± 16.33) mg to (37.80 ± 7.53) mg (mean reduction of 78.87 mg, Cohen’s *d* = 3.2, 95% CI: 2.8–3.6, *P* < 0.001), whereas the NRS score improved from (8.03 ± 1.76) to (1.43 ± 0.55) (Cohen’s *d* = 2.8, 95% CI: 2.4–3.2, *P* < 0.001). QOL scores increased by 173% [from (19.15 ± 3.63) to (52.36 ± 10.78); (Cohen’s *d* = 2.5, 95% CI: 2.1–2.9, *P* < 0.001)], and KPS scores improved from (72.04 ± 13.97) to (97.32 ± 9.68) (Cohen’s *d* = 1.9, 95% CI: 1.5–2.3, *P* < 0.001). Quantitative imaging analysis revealed significant tumor volume reduction from (45.8 ± 12.3) cm³ to (18.6 ± 6.7) cm³ (mean reduction of 27.2 cm³, Cohen’s *d* = 2.1, 95% CI: 1.7–2.5, *P* < 0.001), with mean CT values increasing from (52.4 ± 14.2) HU to (196.3 ± 38.5) HU (mean increase of 143.9 HU, Cohen’s *d* = 2.4, 95% CI: 2.0–2.8, *P* < 0.001), indicating tumor necrosis and osteogenic repair (Table 2).

**Table 2:** OMEC, NRS, QOL, and KPS scores before and after seed implantation in 253 patients with bone metastases.

Time point	OMEC (mg)	NRS	QOL	KPS
Pre-operative	116.67 ± 16.33	8.03 ± 1.76	19.15 ± 3.63	72.04 ± 13.97
1 week after implantation	79.33 ± 20.07	4.95 ± 1.87	25.52 ± 4.95	78.32 ± 15.07
4 weeks after implantation	60.80 ± 14.47	4.07 ± 1.43	31.46 ± 8.36	83.54 ± 10.78
8 weeks after implantation	59.60 ± 16.40	2.75 ± 1.21	47.52 ± 10.01	90.46 ± 13.51
12 weeks after implantation	48.53 ± 9.87	2.09 ± 0.99	50.15 ± 11.33	93.54 ± 14.85
24 weeks after implantation	37.80 ± 7.53	1.43 ± 0.55	52.36 ± 10.78	97.32 ± 9.68

**Fig 2:**
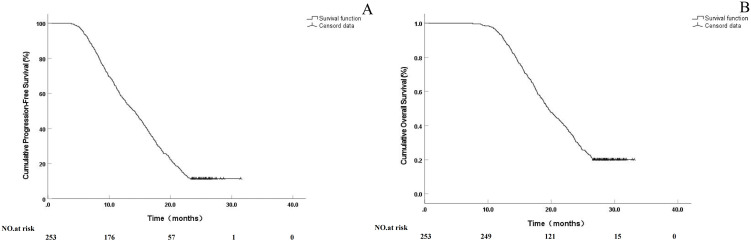
Kaplan-Meier curves for localprogression-free survival (LPFS) and overall survival (OS)‌‌.

**Fig 3:**
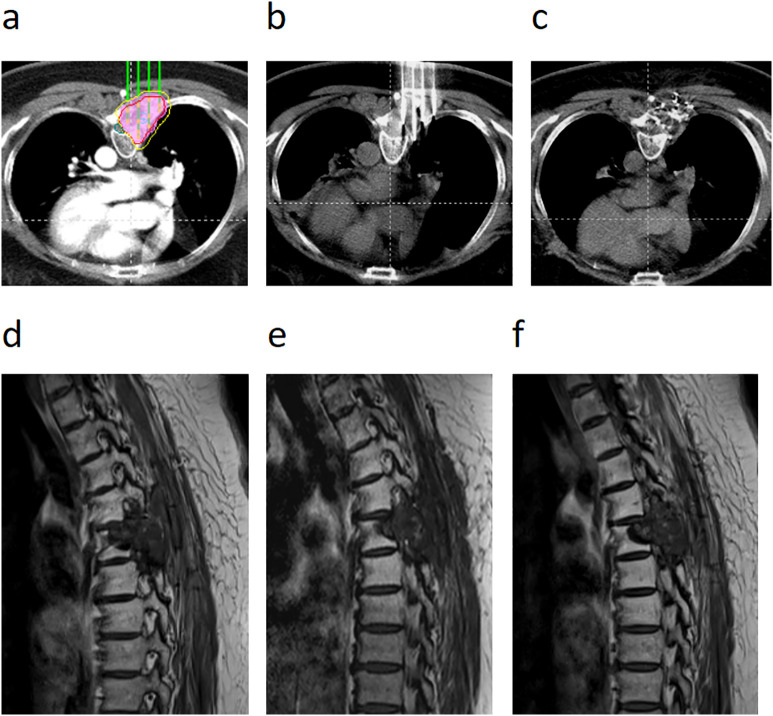
A patient with thoracic spine metastasis after resection of lung cancer. **(a)** Under preoperative enhanced CT guidance, the ^125^I seed implantation system was used for preoperative planning. **(b)** In interventional implantation, imaging after the template-assisted CT-guided needle insertion showed that the needle insertion angle and the depth were good, and the large blood vessels were successfully avoided. **(c)** Immediate post-interventional review; CT revealed a uniform seed distribution in the lesion area. **(d)** Preoperative MRI showed space-occupying lesions in the 4th and 5th thoracic accessory areas as well as the posterior costal region. **(e)** MRI at 1 year after seed implantation showed that the lesion had narrowed. **(f)** MRI 2 years after intervention showed that the boundary of the lesion was clear and the lesion was stable.

**Fig 4:**
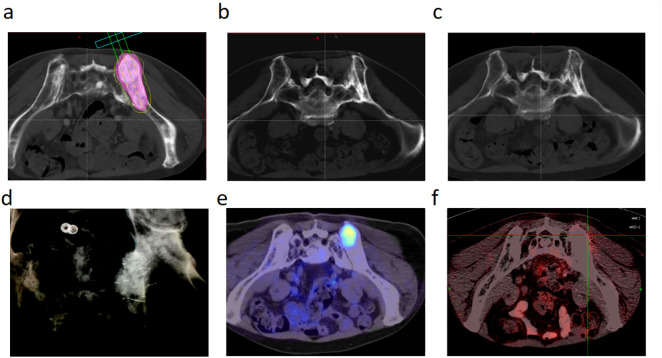
A patient with pelvic metastasis of prostate cancer. **(a)** Preoperative plan of seed implantation designed with enhanced CT according to ^125^I seed implantation system. **(b)** Imaging after template-assisted CT-guided needle insertion showed that the needle insertion angle and the depth were good, and no significant displacement of the puncture needle was noted during the intervention. **(c)** Review immediately after the intervention; CT showed uniform division of seeds in the area of the lesion. **(d)** Re-examination 4 days after intervention confirmed that the seeds were well located within the lesion. **(e)** Preoperative PET-CT showed right pelvic metastasis and radiopharmaceutical aggregation in the metastatic lesion. **(f)** PET-CT 2 years after radioactive ^125^I seed brachytherapy showed no radiopharmaceutical aggregation in the right pelvic metastatic lesion.

Tumor response evaluation according to RECIST 1.1 criteria revealed an ORR of 42.29% and DCR of 79.05% in 1 month, improving to 69.56% and 88.93%, respectively, in 6 months. Furthermore, 15 patients (5.9%) with osteoblastic or mixed-type metastases achieved a complete response at the 6-month assessment. The median follow-up time for the entire cohort was 24.5 months (range: 7.6–33.1 months). At the time of analysis, 85 patients (33.6%) were alive and 168 (66.4%) had died. The median LPFS was 14.1 months (95% CI: 12.6–15.5), whereas OS was 19.5 months (95% CI: 18.2–20.8). The 1-year and 2-year survival rates were 92.9% and 30.0%, respectively, with no bone metastasis-specific mortality recorded. Regarding safety, one case (0.39%) of transient paraplegia was reported. The symptoms developed 48 h after the procedure and were most likely attributable to localized edema rather than hemorrhage or direct radiation injury. The patient received intravenous corticosteroid therapy, with complete resolution of symptoms within 7 days (Fig 2). Typical cases are illustrated in Figs 3 and 4 (S1 and S2 Figs).

### Safety evaluation

The treatment demonstrated a favorable safety profile with predominantly mild-to-moderate adverse events. Transient local pain was the most common adverse effect, occurring in 67.19% (170/253) of patients, with 99 cases (39.13%) graded as CTCAE grade 1 and 71 (28.06%) as grade 2. Other observed complications included subcutaneous hematoma in 19.76% (50/253), skin reactions in 4.35% (11/253), and seed displacement in 6.32% (16/253) of patients, all of which were CTCAE grade I–II. One case (0.39%) of transient paraplegia was reported, which resolved within 1 week with appropriate management. No grade III–IV adverse events or serious complications such as myelosuppression, needle tract seeding, pneumothorax, or uncontrolled hemorrhage were observed during the study period (Table 3). All adverse events were effectively managed with symptomatic treatment, demonstrating the acceptable safety profile of the procedure for patients with refractory bone metastasis.

**Table 3:** Adverse reactions after seed implantation in 253 patients with refractory bone metastases.

Adverse Reactions	Grade I	Grade II	Grade III	Grade IV
Local pain	99	71	0	0
Subcutaneous hematoma	35	15	0	0
Skin reaction	11	0	0	0
Seed displacement	14	2	0	0
Paraplegia	1	0	0	0

### Immunological changes

**Fig 5:**
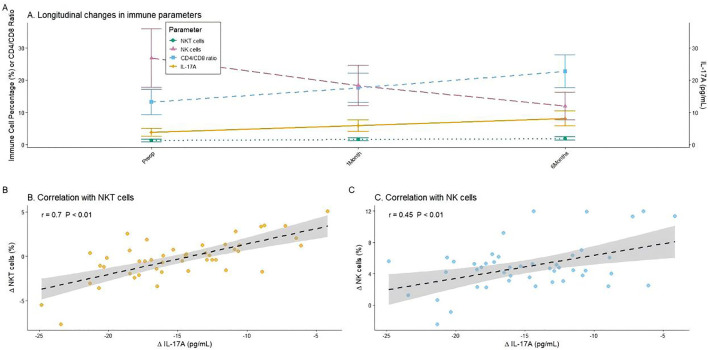
Immunomodulatory effects of ^125^I seed brachytherapy. A: Longitudinal changes in immune cell subsets and IL-17A levels. Data are presented as mean ± standard error of the mean. **(B and C)** Correlation analysis between the change in IL-17A (ΔIL-17A) levels and the changes in NKT (ΔNKT) cell and NK (ΔNK) cell proportions from baseline to 6 months.

Significant immunomodulation was observed after brachytherapy (partial *η*² = 0.45, 95% CI: 0.38–0.52; repeated-measures ANOVA). Flow cytometry showed progressive increases in the proportions of immune effector cells: the proportion of CD3 ⁺ CD16 ⁺ /CD56 ⁺ NKT cells increased from (3.80 ± 1.20)% preoperatively to (5.90 ± 1.80)% at 1 month and (8.10 ± 2.30)% at 6 months (*P* < 0.01) after the surgery, whereas the proportion of CD3 ⁻ CD16 ⁺ /CD56 ⁺ NK cells increased from (13.20 ± 3.90)% to (17.60 ± 4.50)% and (22.70 ± 5.10)%, respectively (*P* < 0.01). The CD4 ⁺ /CD8 ⁺ ratio also improved gradually from (1.28 ± 0.42) to (1.65 ± 0.48) and (1.92 ± 0.56), respectively (*P* < 0.05). In contrast, serum IL-17A levels decreased progressively from (26.80 ± 9.10) pg/mL to (18.30 ± 6.20) pg/mL and (11.90 ± 4.30) pg/mL, respectively (*P* < 0.001). Negative correlations were found between IL-17A level and proportion of NKT cells (*r* = −0.70, *P* < 0.01) and NK cells (*r* = −0.45, *P* < 0.01), indicating a potential immunoregulatory link (Table 4 and Fig 5).

**Table 4:** Dynamic changes in immune parameters following ^125^I seed implantation.

Immune Parameter	Preoperative	1 Month Postoperative	6 Months Postoperative	P Value
NKT cells (%)	3.80 ± 1.20	5.90 ± 1.80	8.10 ± 2.30	< 0.01
NK cells (%)	13.20 ± 3.90	17.60 ± 4.50	22.70 ± 5.10	< 0.01
CD4 ⁺ /CD8 ⁺ ratio	1.28 ± 0.42	1.65 ± 0.48	1.92 ± 0.56	< 0.05
IL-17A (pg/mL)	26.80 ± 9.10	18.30 ± 6.20	11.90 ± 4.30	< 0.001

## Discussion

Bone metastases are a prevalent complication of advanced malignancies. They frequently cause severe pain, functional impairment, and diminished quality of life, posing a major challenge to clinical palliative care. Although EBRT has long been the standard local treatment for symptomatic bone metastases, approximately 40% of patients fail to achieve satisfactory pain relief. This might be due to tumor radioresistance, anatomical constraints (e.g., proximity to the spinal cord or large blood vessels), or prior radiation exposure. The therapeutic options are often limited for these patients who have failed multiple lines of treatment [14–15]. Based on the clinical data from 253 patients with refractory bone metastases across multiple centers, this study confirmed that 3D-printed template-assisted CT-guided ^125^I seed implantation achieved a 100% technical success rate, and also exhibited unique advantages in terms of symptom control, local tumor control, immunomodulation, and safety, thereby providing a novel solution to this clinical dilemma.

All patients in this study successfully underwent the procedure, with an average operative time of only (33.3 ± 6.8) min. This improvement in efficiency was attributed to the precise positioning capability of the 3D-printed template [16]. Preoperatively, the template customized via CT image reconstruction allowed the pre-planning of puncture channels, thus eliminating the need for repeated intraoperative adjustments of the needle path. This reduced operational difficulty, and also enhanced reproducibility in multicenter applications, which was consistent with the conclusion reported by Xiang et al. that “template-guided technology can reduce the operation time of bone metastasis seed implantation by more than 30%” [17].

In terms of clinical benefits, the median time to pain relief was 3.5 days and the median duration of pain relief reached 10.7 months, comparing favorably with historical reports of EBRT [18–19]. Additionally, the daily OMEC decreased from (116.67 ± 16.33) mg to (37.80 ± 7.53) mg at 24 weeks postoperatively, a reduction of 67.6%. The NRS score decreased from (8.03 ± 1.76) to (1.43 ± 0.55), the QOL score increased by 173%, and the KPS score increased from (72.04 ± 13.97) to (97.32 ± 9.68). These improvements confirmed the advantage of the technology in terms of “rapid symptom relief and functional recovery of patients.” The significant reduction in OMEC alleviated drug-related adverse effects such as constipation and drowsiness, and also lowered the risk of long-term addiction, particularly for patients who previously relied on high-dose opioids but still struggled with pain control. This was consistent with the result reported by Shao et al. that “^125^I seed implantation could reduce opioid consumption by more than 50% in patients with refractory bone metastases” [20]. Radiographic assessments further validated the local efficacy of the treatment. The tumor volume decreased from (45.8 ± 12.3) cm³ to (18.6 ± 6.7) cm³, and the mean CT density value (HU) increased from (52.4 ± 14.2) to (196.3 ± 38.5) at 6 months postoperatively. This change indicated significant tumor necrosis (reduction in the number of low-density areas) accompanied by osteogenic repair (increase in the number of high-density areas), aligning with the imaging principle in solid tumor treatment that “elevated HU values usually correspond to lesion fibrosis or calcification” [21]. According to the RECIST version 1.1, the 6-month ORR and DCR were 69.56% and 88.93%, respectively. These results highlighted the therapeutic benefit of the 3D-printed template-assisted CT-guided approach [22–23], and also suggested a potential advantage over re-irradiation with external beam radiotherapy in this challenging cohort [24].

The most innovative finding of this study was the confirmation that 3D-printed template-assisted CT-guided ^125^I seed implantation induced significant systemic immunomodulatory effects. The proportion of CD3 ⁺ CD16 ⁺ /CD56 ⁺ NKT cells increased from (3.8 ± 1.2)% to (8.1 ± 2.3)%, the proportion of CD3 ⁻ CD16 ⁺ /CD56 ⁺ NK cells rose from (13.2 ± 3.9)% to (22.7 ± 5.1)%, the CD4 ⁺ /CD8 ⁺ T-cell ratio recovered from (1.28 ± 0.42) to (1.92 ± 0.56), and the level of the pro-inflammatory factor IL-17A decreased from (26.8 ± 9.1) pg/mL to (11.9 ± 4.3) pg/mL at 6 months, postoperatively. Moreover, the change in IL-17A levels was significantly negatively correlated with the changes in NKT and NK cell proportions (*r* = −0.70 and −0.45, respectively). This immune remodeling process revealed the underlying mechanism of this treatment beyond “local radiotherapy.” Nevertheless, the immunological findings are hypothesis-generating and require validation in prospective studies with comprehensive immune profiling.

From an immunological perspective, the continuous low-dose radiation emitted by ^125^I seeds activates antitumor immunity through two pathways. First, radiation induces immunogenic cell death (ICD) of tumor cells, releasing tumor-associated antigens (TAAs) and damage-associated molecular patterns (DAMPs), which, in turn, activate innate immune cells such as NKT and NK cells [25]. Second, low-dose radiation enhances T-cell infiltration and activation, contributing to the reversal of tumor microenvironment immunosuppression [26]. The recovery of the CD4^+^/CD8^+^ ratio in this study suggested that radiation promoted the proliferation and activation of effector CD8^+^ T cells, enhancing the cytotoxic immune response. This was consistent with previously reported results, that is, “brachytherapy can increase the proportion of CD8^+^ T cells in the peripheral blood of patients with tumors” [27]. More importantly, the decrease in IL-17A level was negatively correlated with the increase in the proportion of cytotoxic immune cells. This indicated that the treatment inhibited the Th17 cell–mediated pro-inflammatory pathway to reduce bone destruction, while enhancing antitumor immunity, thereby forming a positive cycle of “inhibiting bone metastasis progression-activating immune response.” This provided an immunological basis for explaining why “the duration of pain relief in patients is longer than expected from simple local tumor control.”

Safety is a core prerequisite for treatment selection for patients with refractory bone metastases who have failed multiple lines of treatment. In this study, 67.19% of patients experienced transient local pain of CTCAE grade 1–2, 19.76% developed subcutaneous hematoma, and 6.32% had seed displacement. All adverse events were mild to moderate and resolved with symptomatic treatment. Only one patient (0.39%) developed transient paraplegia, which recovered within 1 week. No severe complication, such as myelosuppression, needle tract seeding, or treatment-related death, was observed. Overall, the adverse events associated with this therapeutic regimen were mild and manageable, demonstrating favorable and reliable clinical safety.

Technically, the application of 3D-printed templates was pivotal for enhancing treatment safety. Preoperatively, adjacent organs at risk were precisely delineated to ensure that the distance between the radioactive seeds and the spinal cord exceeded 1 cm, with a minimum 1-cm separation from the skin. This effectively mitigated radiation-induced damage to normal tissues. Intraoperatively, real-time dose optimization was performed using a brachytherapy treatment planning system, whereas postoperative dose verification was conducted to further ensure the conformity of the radiation dose. These measures collectively reduced the risk of seed migration and the incidence of complications. This technology enabled safe treatment even in anatomically complex regions, thereby providing a viable therapeutic option for refractory patients with poor physical status who could not tolerate aggressive treatment modalities [28].

Compared with the report by Wang et al. that “the 2-year survival rate of ^125^I seed implantation for pelvic bone metastases was 45.5%” [29], the 2-year survival rate in this study was 30.0%, which seemed slightly lower. However, the heterogeneity of the study populations must be considered. Wang et al. included patients mainly with isolated pelvic bone metastases without significant visceral metastasis, whereas in this study, 243 (96.0%) of the 253 patients had lesions with a maximum diameter ≥3 cm, 187 (73.9%) had osteolytic metastases (often indicating more aggressive tumors), and some patients had concurrent visceral metastasis, resulting in more complex baseline conditions. Nevertheless, the median OS of patients in this study was still 19.5 months, and the median LPFS was 14.1 months, indicating that this technology still achieved meaningful survival benefits in patients with more severe conditions.

From a clinical practice perspective, the value of this study was in “relieving symptoms and controlling local metastases” and also in creating a “window period” for subsequent treatment. Patients experienced pain relief and functional improvement by effectively controlling the burden of bone metastases, enabling them to tolerate more aggressive treatment of the primary tumor (e.g., chemotherapy, targeted therapy, and immunotherapy). Thus, this technique served as a crucial bridging strategy in the comprehensive management of patients with refractory bone metastases [30].

Despite being a multicenter retrospective analysis with a large sample size, this study still had certain limitations. First, the retrospective design might have led to selection bias, such as information bias. For example, although the definition of “refractory” was established referring to extensive literature, slight differences might have existed in judgment criteria among various centers. Second, patients had diverse primary tumor types, and the biological behavior of different tumors might have impacted treatment response; however, this study did not include subgroup analyses. Most importantly, the lack of a concurrent control group (e.g., comparison with re-irradiation using EBRT or ablation therapy) prevented direct confirmation of the comparative efficacy or superiority of this technique.

Future research could be directed in three directions. First, prospective randomized controlled trials should be conducted to compare the efficacy and safety of 3D-printed template-assisted CT-guided ^125^I seed implantation with re-irradiation with EBRT, thereby clarifying its position in the treatment sequence. Second, subgroup analyses should be performed based on different primary tumor types and metastasis sites to explore efficacy predictors. Third, multiomics technologies should be combined to deeply analyze the immunomodulatory mechanism of this technology, thus providing a theoretical basis for combined immunotherapy.

In conclusion, 3D-printed template-assisted CT-guided ^125^I seed implantation was a safe and effective method for treating refractory bone metastases. Its advantages included rapid and long-lasting pain relief, excellent local tumor control, tolerable safety, and unique immunomodulatory effects. This technology overcame the limitations of traditional treatments (e.g., EBRT and surgery) in refractory cases, and also created favorable conditions for subsequent treatment of the primary tumor, thereby providing a new therapeutic option for the comprehensive management of patients with bone metastases from advanced cancer. However, future high-quality prospective studies are needed to further validate its efficacy and promote its broader clinical application.

## Supporting information

S1 Fig Figure (a-e) showed the actual distribution of seeds and the dose in target volume after seed implantation; Figure (f) showed the dose-volume histograms of gross tumor volume postoperation. (TIF)

S2 Fig Figure (a-e) showed the actual distribution of seeds and the dose in target volume after seed implantation; Figure (f) showed the dose-volume histograms of gross tumor volume postoperation. (TIF)

## Abbreviations

OMEC: oral morphine equivalent consumption
NRS: numerical rating scale
QOL: quality of life
KPS: karnofsky performance status
HU: hounsfield unit
RECIST: response evaluation criteria in solid tumors
ORR: objective response rate
DCR: disease control rate
NKT: natural killer T
LPFS: local progression-free survival
OS: overall survival
EBRT: external beam radiotherapy
TPS: treatment planning system
CTV: clinical target volume
PTV: planned target volume
CTCAE: common terminology criteria for adverse events
IRB: institutional review board
ICD: immunogenic cell death
TAAs: tumor-associated antigens
DAMPs: damage-associated molecular patterns
Treg: regulatory T cells
BTPS: brachytherapy treatment planning system
