# Supplementary figures and images for: The radioactive 3D-printed template-assisted CT-guided 125I seed implantation for refractory bone metastases: A multicenter retrospective analysis of efficacy, safety, and immune function changes
Source: PLoS One. 2026 May 11;21(5):e0347893. doi: 10.1371/journal.pone.0347893 (PMC13160349; doi:10.1371/journal.pone.0347893)

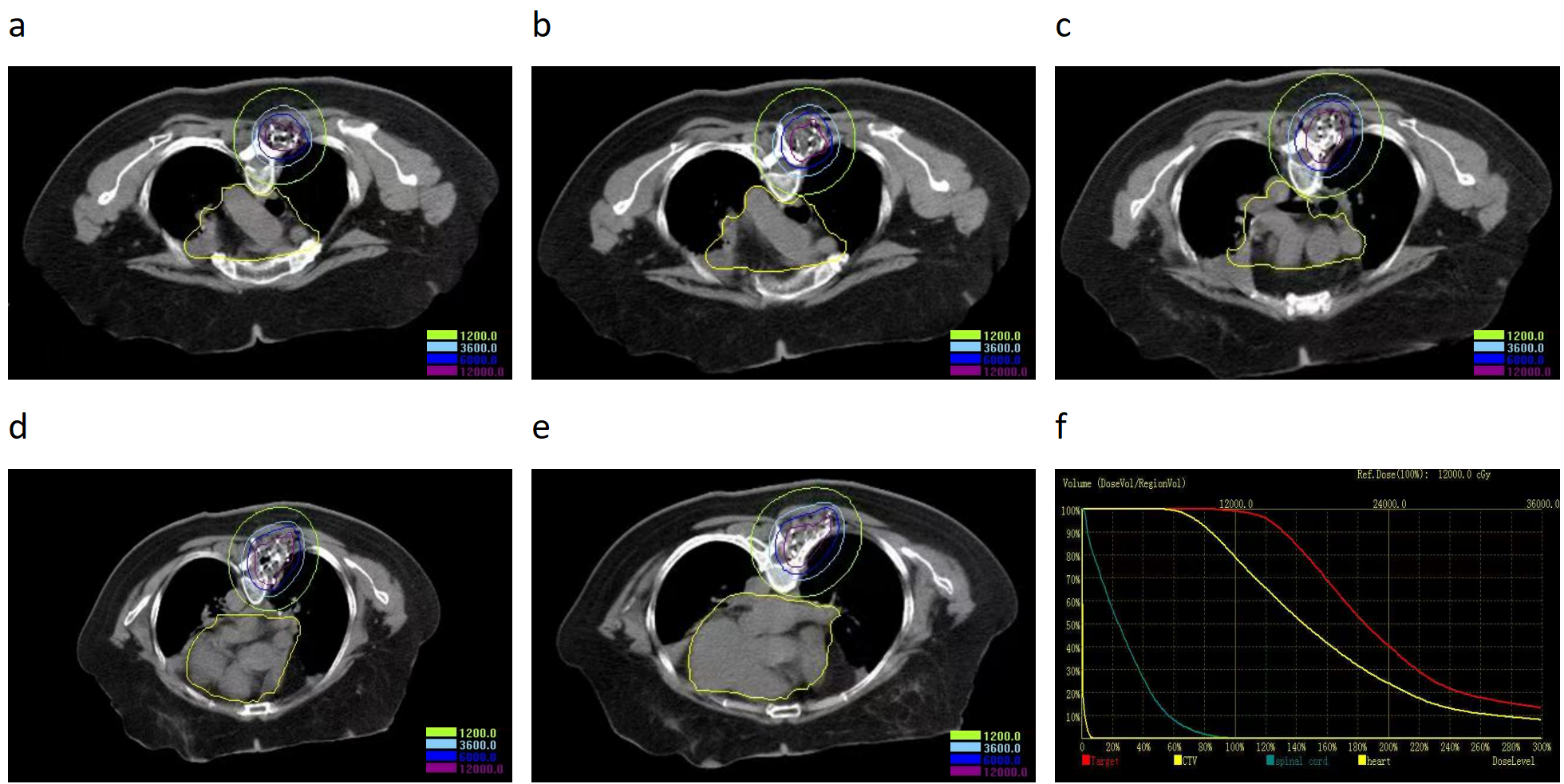

Supplement: S1 Fig — (TIF) [file pone.0347893.s001.tif]

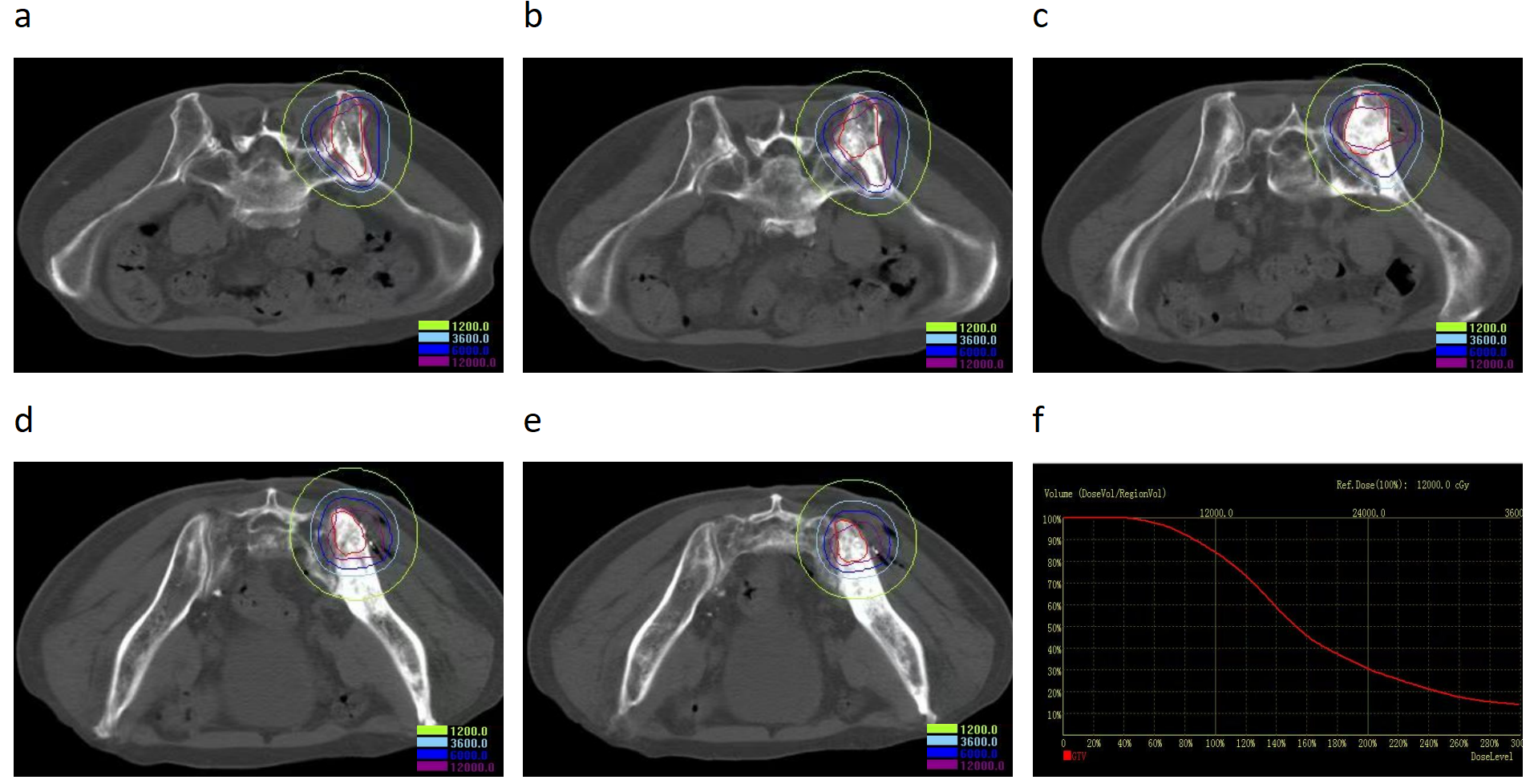

Supplement: S2 Fig — (TIF) [file pone.0347893.s002.tif]
